# Progress and prospects of nanozymes for enhanced antitumor therapy

**DOI:** 10.3389/fchem.2022.1090795

**Published:** 2022-12-02

**Authors:** Yulong Yu, Weiheng Zhao, Xianglin Yuan, Rui Li

**Affiliations:** Department of Oncology, Tongji Hospital, Tongji Medical College, Huazhong University of Science and Technology, Wuhan, China

**Keywords:** nanozymes, catalytic therapy, enzyme mimics, cancer therapy, reactive oxygen species

## Abstract

Nanozymes are nanomaterials with mimicked enzymatic activity, whose catalytic activity can be designed by changing their physical parameters and chemical composition. With the development of biomedical and material science, artificially created nanozymes have high biocompatibility and can catalyze specific biochemical reactions under biological conditions, thus playing a vital role in regulating physiological activities. Under pathological conditions, natural enzymes are limited in their catalytic capacity by the varying reaction conditions. In contrast, compared to natural enzymes, nanozymes have advantages such as high stability, simplicity of modification, targeting ability, and versatility. As a result, the novel role of nanozymes in medicine, especially in tumor therapy, is gaining increasing attention. In this review, function and application of various nanozymes in the treatment of cancer are summarized. Future exploration paths of nanozymes in cancer therapies based on new insights arising from recent research are outlined.

## Introduction

Cancer is the main cause of death and has a significant negative impact on both the economy and quality of life (Sung et al., 2021). Although conventional cancer treatment strategies such as radiotherapy, chemotherapy, and immunotherapy have been developed and validated for various types of cancers, the clinical efficacy of these therapies is still restricted. Drug tolerance, toxic side effects, radiation resistance and immune evasion remain formidable obstacles in cancer therapy. As the incidence and mortality of malignancies continue to rise, novel therapeutic agents have long been sought by scientists (Bray et al., 2021).

Enzymes are proteins or nucleic acids synthesized in an organism as a biological catalyst. They can be involved directly or indirectly in a variety of life processes in organisms, including cell metabolism, proliferation, differentiation, and aging. Enzymes have also been shown to contribute to the emergence of diseases. Tyrosinase, for instance, is required for the body to synthesize melanin and its absence can cause albinism (Song et al., 2018; Michaud et al., 2022). Numerous lung illnesses, including asthma and chronic obstructive pulmonary disease, have been linked to nitric oxide synthases (Scott et al., 2021). It has been demonstrated that matrix metalloproteinases, which are overexpressed in tumor cells, facilitate cancer metastasis (Shi et al., 2015; Chen et al., 2020). However, protease and ribonuclease easily and quickly break down natural enzymes, making it challenging to store and transport them. On the other hand, the use of enzymes in therapeutic settings is constrained by the strict conditions that natural enzyme catalysis requires, such as a particular pH and temperature.

With the rapid development of nanotechnology, nanomaterials which can be manipulated on atomic and molecular scale are applied in medicine (Chai et al., 2022; Wang et al., 2022c; Zhang et al., 2022d; Farheen et al., 2022; Yang et al., 2022). Novel nanomaterials have demonstrated outstanding potentials in clinical applications with diverse functions (Filik et al., 2021; Li et al., 2022b; Kim et al., 2022). Nanozymes are artificial nanomaterials that exhibit intrinsic catalytic properties similar to that of natural enzymes and are attracting a massive attention (Jiang et al., 2019; Liang and Yan, 2019). To date, substantial nanomaterials have been discovered to possess enzyme-like activities, including carbon-based, metal-based, metal oxide-based and metal chalcogenide nanozymes (Robert and Meunier, 2022). By modifying structural composition and surface properties, nanozymes with different enzyme-like activities can be obtained, catalyzing the conversion of substrates into products and speeding up biological reactions under appropriate physiological circumstances. In contrast to natural enzymes, nanozymes possess the advantages of high stability, simplicity of modification, targeting ability, and low cost (Wang et al., 2019a). As a result, the application trend of nanozymes in tumor treatment has become a hotspot. However, there are few reviews on nanozymes for antitumor therapies.

**SCHEME 1:**
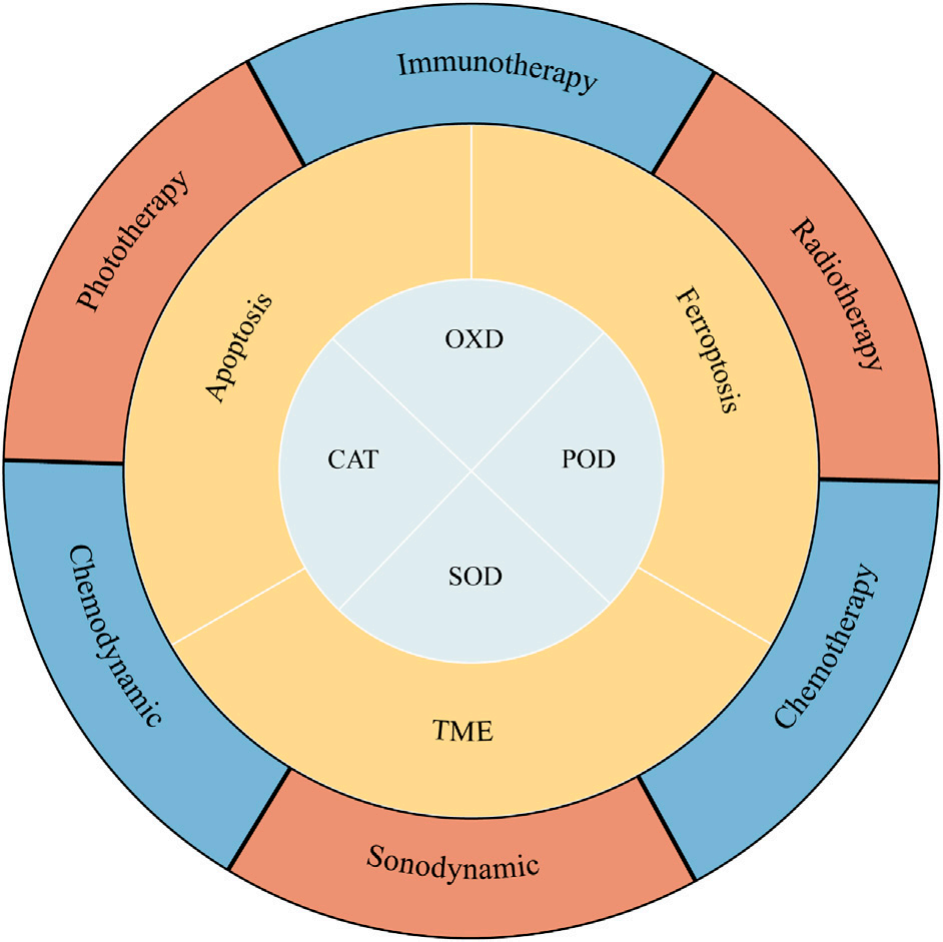
Nanozymes are engineered to enhance multiple antitumor therapy.

In this review, the recent research achievements and progress of nanozymes in cancer treatment are highlighted (Scheme 1). Firstly, we summarize the mechanisms underlying the most common antitumor effect of nanozymes. Then the promising applications and defects of nanozyme-based synergistic antitumor strategies are discussed.

## Antitumor mechanisms of nanozymes

**FIGURE 1:**
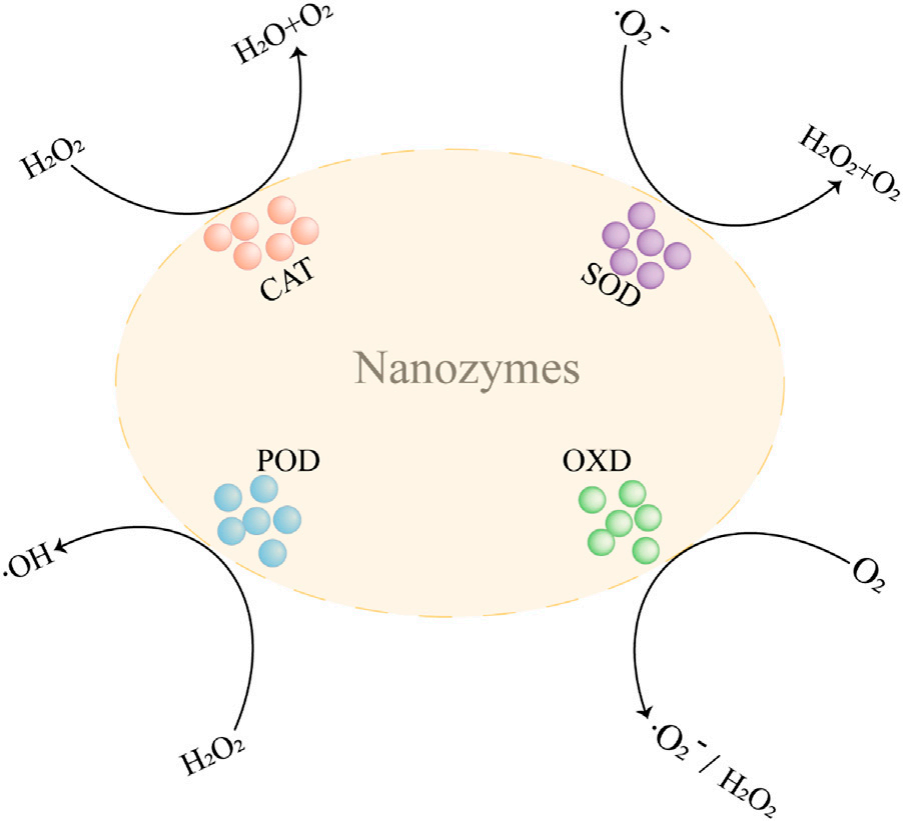
The catalysis reaction mediated by nanozymes. CAT, catalase; POD, peroxidase; OXD, oxidase; SOD, superoxide dismutase; H_2_O_2_, hydrogen peroxide; O_2_, oxygen; •OH, hydroxyl radicals; •O_2_^−^, superoxide radicals.

**FIGURE 2:**
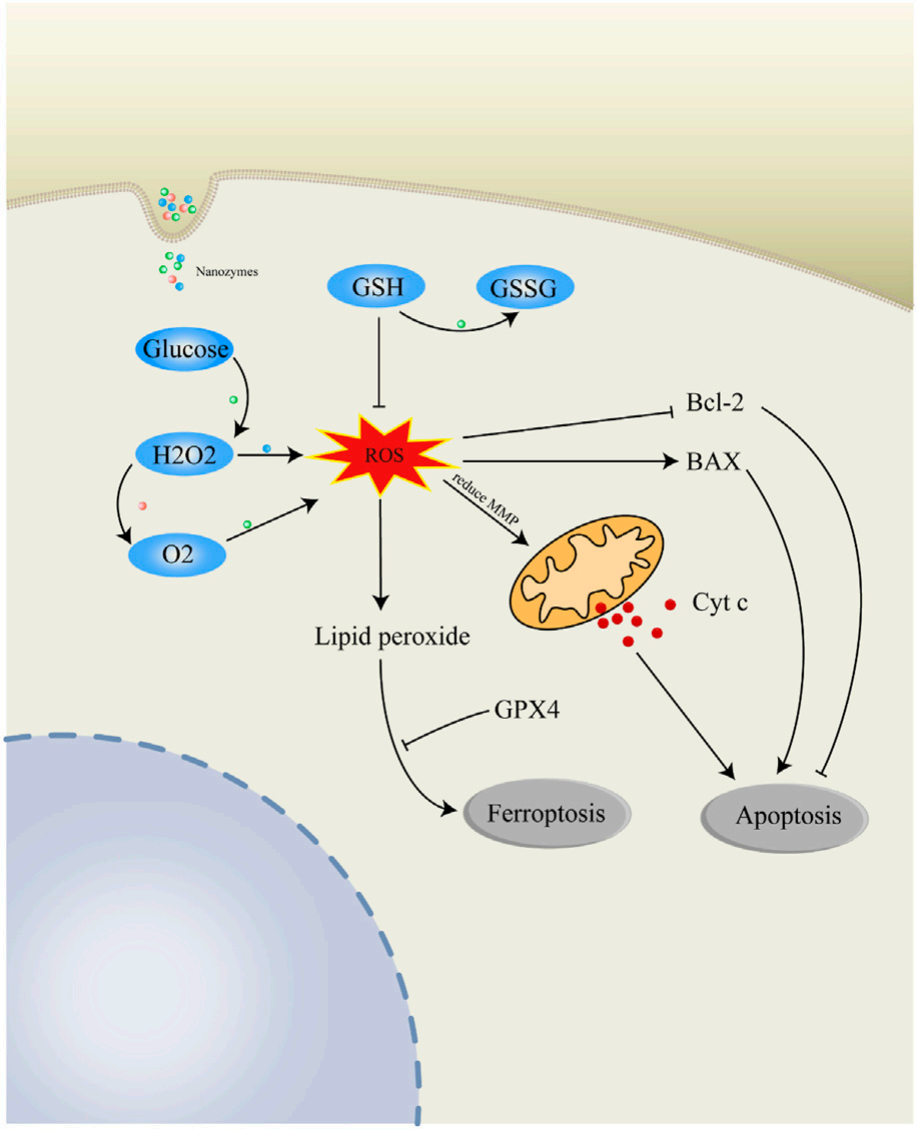
Anticancer mechanisms of nanozymes. Catalase (CAT)-like nanozymes can catalyze the decomposition of H_2_O_2_ and product O_2_ for the alleviation of hypoxia in TME. Peroxidase (POD)-like nanozymes can promote the degradation of H_2_O_2_ to produce highly toxic ROS. Oxidase (OXD)-like nanozymes can metabolize O_2_ to produce ROS. Nanozymes with glucose oxidase activity can promote the oxidation of glucose and generate H_2_O_2_ for further reaction. GSH can deplete intracellular ROS and be consumed by nanozymes to generate GSSG. Excessive intracellular ROS regulate mitochondria-related Bcl-2/BAX/Cyt c apoptosis pathway. Highly active ROS can form lipid peroxide which can induce ferroptosis of cells. ROS, reactive oxygen species; H_2_O_2_, hydrogen peroxide; O_2_, oxygen; GSH, glutathione; GSSG, glutathione disulfide; Cyt c, cytochrome c; Bcl-2, apoptosis regulator Bcl-2; BAX, apoptosis regulator BAX; GPX4, phospholipid hydroperoxide glutathione peroxidase.

In recent decades, Nanozymes in oncology are compelling hotspots. With the advancement of material science, the design and synthesis of nanozymes is becoming more and more sophisticated (Jiang et al., 2019). Due to their excellent performance, nanozymes have developed into powerful tools for the treatment of tumors. Nanozymes that are more widely used in tumor therapy are peroxidase (POD), oxidase (OXD), catalase (CAT), and superoxide dismutase (SOD) (Figure 1). In this section, we discuss the anticancer mechanisms of nanozymes based on the recent reports (Figure 2).

### Apoptosis induction

Apoptosis as a programmed cell death maintains the balance between cell death and cell proliferation, regulating the process of carcinogenesis. One of the characteristics of cancer is evasion of apoptosis, which makes cancer cells live with the ability to keep proliferating and escape cell death (Hanahan and Weinberg, 2011). Due to this characteristic of cancer, apoptosis induction becomes an effective approach for cancer therapy, which can kill malignant cells with no activation of an inflammatory response and little side effects to healthy cells (Chaudhry et al., 2022).

Nanozymes with multiple enzymatic activities can effectively produce reactive oxygen species in the tumor microenvironment through cascade catalytic reactions, thereby promoting the apoptosis of cancer cells (Zhao et al., 2021b; Yao et al., 2022b; Wang et al., 2022f). Ma et al. (2022) designed nanozymes with three nanoceria structures including nanoceria-cube, nanoceria-poly and nanoceria-rod. Among them, nanoceria-rod with highest concentration of surface oxygen vacancies has the highest POD-like and OXD-like enzyme activity. Due to the suitable isoelectric point, nanoceria-rod nanozyme can selectively enter the lysosomes and phagosomes of tumor cells in acidic environments. Then a large amount of ROS is produced and mitochondrial membrane potential is reduced, resulting in malignant cell apoptosis with no toxicity to normal cells.

Typically, the mechanism of nanozymes to induce apoptosis is mainly *via* mitochondrial apoptotic pathway. For example, PP-MnO(x) NPs, an OXD-like nanozyme, can catalyze O_2_ to produce abundant ROS and induce the decrease of mitochondrial membrane potential. Meanwhile, PP-MnO(x) NPs activate BAX and Caspase-3, as well as decrease the expression of Bcl-2 (Xi et al., 2021). Moreover, Chu et al. (2020) Synthesized M@AAO@HFe–TA, a nanozyme system with amino acid oxidase (AAO) and POD activity. M@AAO@HFe–TA system induced apoptotic in tumor due to amino acid starvation and cascade Fenton reaction effect. The expression of Bcl-2 was suppressed and the level of BAX was increased, releasing the cytochrome C (cyt C) to the further active caspase 3. These suggest that nanozymes induce ROS generation and oxidative stress to initiate mitochondrial-related apoptosis.

### Ferroptosis induction

Ferroptosis is a type of iron-dependent non-apoptotic cell death related to abnormal level of intracellular ferrous iron. Recently, ferroptosis was found to be widely present in many cancers with characteristics of mitochondrial damage and plasma membrane disrepair (Chen et al., 2021b; Tang et al., 2021). The induction of ferroptosis can potentiate the efficacy of chemotherapy, radiotherapy and immunotherapy in drug-resistant cancer (Friedmann Angeli et al., 2019; Ye et al., 2020; Fan et al., 2021), suggesting that targeting ferroptosis strategy is a viable method for cancer treatment (Mou et al., 2019).

Ferroptosis is mainly provoked by ROS accretion and unrestricted lipid peroxidation, which is induced by excessive endogenous Fenton reaction and the exhaustion of GSH (Wei et al., 2020; Yang et al., 2021). Thus, nanozymes with peroxidase-like and glutathione peroxidase-like enzyme activity are designed as ferroptosis inducer for killing cancer cells (Meng et al., 2021). Zhang et al. (2022b) fabricated gemcitabine (Gem)-loaded carbonaceous nanoparticles (MFC-Gem) as peroxidase-like and glutathione oxidase-like nanozymes. The dual-activity MFC-Gem nanozyme effectively catalyzes the Fenton reaction to generate huge ROS and deplete GSH for oxidative damage. Meanwhile, glutathione peroxidase 4 (GPX4), a lipid repair enzyme which can use GSH to detoxify lipid peroxidation, loss activities in MFC and MFC-Gem treated mice, making cancer cells more susceptible to ferroptosis. Similarly, boron and nitrogen cooped graphdiyne (BN-GDY) as a metal-free nanozyme possesses capability to produce •OH in the presence of H_2_O_2_ and boost GSH depletion, setting the onset of ferroptosis. Interestingly, unlike the most reported enzymes, the catalytic reaction of BN-GDY undergoes a symbolic sequence Bi−Bi mechanism instead of a ping-pong Bi−Bi mechanism (Zhang et al., 2022a). Moreover, Fenton reaction-independent ferroptosis catalyzed by photothermal nanozyme is a novel insight for ferroptosis-related therapy, due to its satisfactory therapeutic effects in tumor therapy (Xing et al., 2021).

### Tumor microenvironment regulation

Due to uncontrolled cell proliferation, aberrant cancer cell metabolism, and abnormal blood vessel development, tumor microenvironment (TME) is generally characterized by hypoxia (Semenza, 2000). The hypoxic TME make tumor cells exhibit increased drug efflux and lessen ROS-induced DNA damage, resulting in strong drug tolerance and radiation resistance (Brizel et al., 1999; Brown and Wilson, 2004; Gacche and Assaraf, 2018; Li et al., 2022a; Li et al., 2022c; Wang et al., 2022e). Nevertheless, the intrinsic hypoxia in TME suppresses the infiltration of anti-tumor immune cells and even induces the polarization of macrophages and T cells to pro-tumor subtype, such as M2 macrophages and regulatory T cells (Zhang et al., 2022c). Therefore, hypoxic TME hinders the efficacy of immunotherapy and augmenting O_2_ supply becomes urgent to improve cancer treatment (Kopecka et al., 2021). Nanozymes with CAT-mimic activity can transfer H_2_O_2_ to O_2_ and supply enough O_2_ to reverse hypoxia tumor microenvironment. Combined with GOx-like nanozymes which consume glucose and produced H_2_O_2_, self-supply O_2_ system is proposed by scientists (Yu et al., 2022).

Yu et al. (2022) modified carbon nitride (C_3_N_4_) with polydopamine before coating it with MIL-100, Gox, and hyaluronic acid to create a PCMGH nanozyme system. C_3_N_4_, as a water-splitting material, catalyzes H_2_O to generate O_2_, whereas MIL-100 acts like POD to raise the quantity of ROS. Upon 808 nm irradiation, polydopamine acts as a photothermal agent which convert light energy into hyperthermia to achieve photothermal therapy. The self-supplying O_2_ and photothermal effect work synergically to improve the performance of chemotherapy and phototherapy.

The mesoporous silica nanorod was served as nanoplatform to integrate MnO_2_ and Au nanoparticles, forming a new biomimetic nanozyme with dual enzyme activities. By decomposing H_2_O_2_, MnO_2_ can catalyze the production of O_2_, displaying enhanced CAT-like activity. Meanwhile, the Au nanoparticles which exhibit GOx-like activity effectively accelerate the oxidation of glucose and provide enough H_2_O_2_ for more O_2_ production. The alleviation of hypoxic environment surrounding tumor sensitizing tumor cells to radiation therapy and photothermal therapy (Yang et al., 2020).

In addition to directly enhance the efficacy of antitumor treatment such as chemotherapy, phototherapy and radiotherapy, increasing O_2_ level *in vivo* can change the expression of genes linked with tumor metabolism, proliferation and metastasis *via* regulating hypoxia-inducible factor 1 (HIF-1) pathway. Au-Pt nanozymes are reported to relieve the hypoxic TME and suppress the expression of HIF-1α, thus reducing lung metastasis (Shen et al., 2022).

## Nanozyme-based enhanced cancer therapies

Though traditional treatments for cancer such as chemotherapy, radiotherapy and Immunotherapy have been widely practiced. Low response rate, drug resistance and toxic side effects still are obstacles that have not yet been overcome. In recent years, nanozymes are employed in multiple cancer treatments due to their various mimic enzyme activities. In this section, we focused on the synergy of nanozymes and conventional cancer therapy (Table 1).

**TABLE 1:** Nanozymes applied in cancer therapies.

Nanozyme	Activity	Mechanism	Application	References
Single-enzyme activity				
PP-MnO(x)	OXD	generate ROS/induce apoptosis	chemotherapy	Xi et al. (2021)
BN-GDY	POD	deplete GSH/induce ferroptosis	catalytic therapy	Zhang et al. (2022a)
Ce_6_/Ftn@MnO_2_	CAT	O_2_ supply	photodynamic therapy	Zhu et al. (2022b)
ABTS@PAH-CNts	POD	photothermal effect/tumor target	photothermal therapy	Chen et al. (2021c)
Ni_0.5_Fe_0.5_S_2_	POD	generate ROS	photothermal therapy/chemodynamic therapy/photodynamic therapy	Zeng et al. (2022a)
RGD-BPNS@SMFN	CAT	O_2_ supply/tumor target	photothermal therapy/photodynamic therapy	Song et al. (2022)
ChA CQDs	OXD	deplete GSH/immune cells infiltration	immunotherapy	Yao et al. (2022a)
PHCNs	POD	induce apoptosis/activate CART cells	immunotherapy	Zhu et al. (2021)
GDY–CeO_2_	CAT	O_2_ supply/radiosensitizer	radiotherapy	Zhou et al. (2021a)
Mn-Ag_2_Se-RGD-PEG	CAT	O_2 S_upply/tumor target	radiotherapy	Wang et al. (2021)
DGZ	GOX	acidification of glucose/generate ROS	chemotherapy	Huo et al. (2022)
Pt-CuS-TAPP	CAT	O_2_ supply	sonodynamic therapy	Liang et al. (2019)
multi-enzyme activity				
AuNPs@N-HCNs	POD, OXD	generate ROS/induce apoptosis	photothermal therapy	Wang et al. (2022f)
MnMoOx	OXD, CAT	O_2_ supply/generate ROS	photothermal therapy	Zhang et al. (2022f)
Au-FeSAzyme	POD, GOD	consume glucose/generate ROS	photothermal therapy/chemodynamic therapy	Feng et al. (2022)
SSMA/DOX	POD, GOD	consume glucose/generate ROS	chemodynamic therapy/chemotherapy	Zheng et al. (2022)
DOX@HMSN/Mn_3_O_4_R	OXD, CAT	deplete GSH/generate ROS	radiotherapy/chemotherapy	Yuan et al. (2022)
PCMGH	GOx,POD	O_2_ supply/generate ROS/photothermal effect	chemotherapy/phototherapy	Yu et al. (2022)
MSNR@MnO2–Au	CAT/GOx	O_2_ supply/generate ROS	radiation therapy/photothermal therapy	Yang et al. (2020)
M@AAO@HFe–TA	POD, AAO	amino acid starvation/Fenton reaction	chemodynamic therapy	Chu et al. (2020)
MFC-Gem	POD, OXD	generate ROS/induce ferroptosis	chemotherapy	Zhang et al. (2022b)
CuPP	POD/CAT/GPx	generate ROS/deplete GSH	immunotherapy	Zeng et al. (2022b)
CDSDM	POD/CAT	O_2_ supply/regulate TME	immunotherapy	Wang et al. (2019b)
Fe_3_O_4_/Pt-FLU	POD/CAT	generate ROS and O_2_/drug deliver	chemotherapy	Nie et al. (2022)
AIMP	CAT/OXD	O_2_ supply/Deplete GSH	sonodynamic therapy	Liu et al. (2021b)

### Nanozymes in phototherapy

**FIGURE 3:**
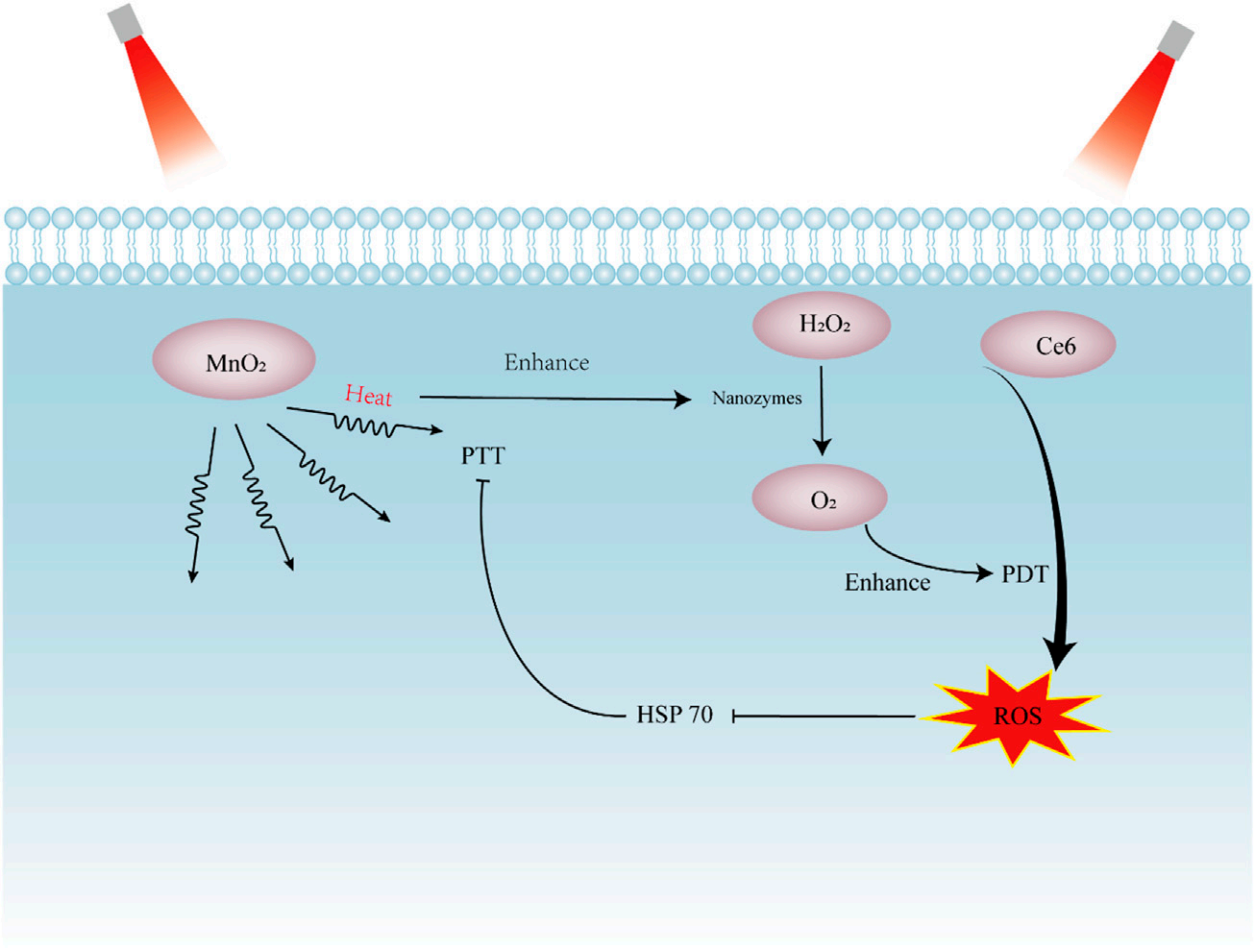
Schematic presentation showing the synergistic effect of nanozymes in photothermal therapy (PTT) and photodynamic therapy (PDT). The photosensitizer Ce6 absorb light and generate cytotoxic ROS. MnO_2_ act as photothermal agents to converts absorbed light into heat and kill tumor cells *via* hyperthermia. PDT decreases the expression of HSP70 to increase sensitivity of tumors to PTT. The heat produced by PTT can enhance the activity of nanozymes, augmenting PDT effect. Ce6, chlorin e6; MnO_2_, manganese dioxide; HSP 70, heat shock protein 70; ROS, reactive oxygen species.

Phototherapies including photothermal therapy (PTT) and photodynamic therapy (PDT) are considered as novel strategies employed widely in tumor treatment, due to their advantages of highly selective, negligible side effects, and minimally invasive (Vieyra-Garcia and Wolf, 2021). PDT provides oxidative damage on the tumor primarily through singlet oxygen, which is converted from triplet molecular oxygen by the photosensitizer as part of the photoconversion reaction (Zheng et al., 2022). While PTT initiates tumor cell death predominantly through hyperthermia *via* plasmonic dissipation, which is converted by the energy of photons absorbed on the photothermal agent under near infrared (NIR) laser irradiation (Figure 3). However, strict conditions such as oxygen leakage, acidic condition and overexpression of GSH in the tumor microenvironment (TME) constrain the photothermal conversion efficacy and ROS production of Phototherapies (Song et al., 2022). Photosensitive nanozymes which can remodel the restricted environment in tumor are intensively studied by scientists to achieve better phototherapeutic efficiency (Wang et al., 2022a; Tang et al., 2022).

In fact, the ROS generation in PDT often ceases with oxygen depletion. To solve this dilemma, an intelligent nanoplatform Ce_6_/Ftn@MnO_2_ with the ability of O_2_ self-supply was constructed, which can reverse unfavorable hypoxia conditions and decrease the expression of hypoxia-inducible factor (HIF)-1α. With adequate O_2_, Ce6/Ftn@MnO_2_ produced a significantly higher cytotoxic ^1^O_2_ amount. The enhanced photodynamic effect of Ce_6_/Ftn@MnO_2_ caused reduction of mitochondrial membrane potential and disruption of lysosome integrity, resulting in tumor cell death (Zhu et al., 2022b). To achieve profound photothermal therapeutic efficacy and minimized side effects, a “dual lock-and-key” type tumor-specific nanozyme (ABTS@PAH-CNts) was designed with higher selectivity. Activated under both H_2_O_2_ and acidic pH, ABTS@PAH-CNts present remarkable photothermal effect, with negligible off-target hyperthermic damage to normal tissues (Chen et al., 2021c).

It is worth noting that PTT can be used to integrate with PDT to get better synergistic antitumor effect. For instance, Ni_0.5_Fe_0.5_S_2_ efficiently converts near-infrared light energy into heat for realizing the photothermal therapy. Moreover, the peroxidase-like property of Ni_0.5_Fe_0.5_S_2_ triggers formation of cytotoxic •OH and ^1^O_2_ to further enhance the efficiency of PDT (Zeng et al., 2022a). Similarly, Song et al. (2022) generated a PTT-PDT dual mode nanoplatform of RGD-BPNS@SMFN, which had self-synergetic behavior and enhanced photonic response than single PDT and PTT. The key self-synergetic mechanism is the PTT promoted temperature-dependent CAT-like activity, which alleviates the hypoxia microenvironment and further encourages the PDT behavior. Indeed, PTT induced hyperthermic temperature can accelerate enzyme-catalyzed ROS generation, which enhances the further PDT therapeutic performance (Zhou et al., 2022). On the other hand, ROS produced by PDT decreases the expression of heat shock protein 70 (HSP70), which was found to protect cells from the damage of heat, thus augmenting photothermal effect (Sun et al., 2021b). Taken together, the combination of PTT and PDT achieves strikingly therapeutic efficacy.

### Nanozymes in immunotherapy

In recent decades, immunotherapy is a hot topic in cancer therapy which revolutionizes cancer treatment because of its striking tumor ablation effect. Multiple immunotherapeutic strategies, including cancer vaccines, immune checkpoint blockade (ICB), and adoptive cell transfer, have been developed by scientists (Rudd, 2019; He and Xu, 2020). However, the application of cancer immunotherapy is limited by low response rates and individual differences mainly due to immunosuppressive TME and heterogenicity. Nanozymes can act as a modulator to reverse immunosuppressive TME by degrading immunosuppressive molecules, inducing infiltration of anti-tumor immune cells and repolarizing pro-tumor cells (Wang et al., 2022b). Thus, nanozymes can be utilized as adjuvant therapeutics to boost immunotherapy efficacy.

**FIGURE 4:**
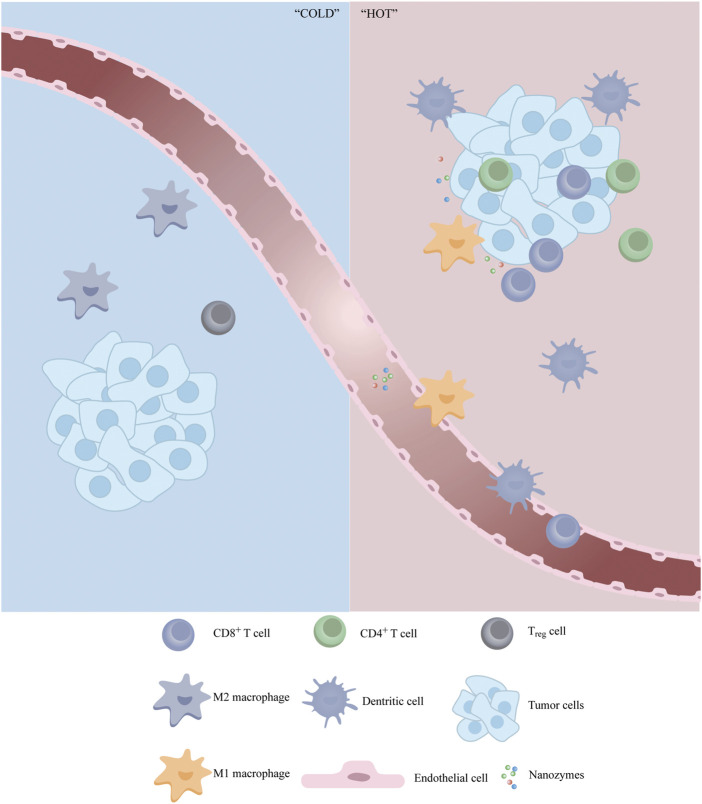
Schematic illustration of the synergistic effect of nanozymes in immunotherapy. Nanozymes can act as modulator to reverse immunosuppressive TME by inducing infiltration of anti-tumor immune cells and repolarizing pro-tumor cells.

The ChA CQDs nanozyme, formed by carbon quantum dots and chlorogenic acid, was reported with GSH oxidase-like activities and induction of cell ferroptosis. In addition to directly inducing cancer cell death through catalytic therapy, ChA CQDs activated the tumor immune microenvironment and converted the “cold tumor” to “hot tumor” by recruiting CD4^+^/CD8^+^ T cells, macrophages, and NK cells (Figure 4). The increase in immune cells provides an important basis for subsequent immunotherapy, changing the situation that immunotherapy is ineffective in several types of cancer (Yao et al., 2022a).

A novel nanozyme (CuPP) with trienzyme-like activities has been proposed to efficiently modulate immunosuppressive TME by releasing O_2_ and •OH but consuming glutathione (GSH). With the reversion of hypoxic microenvironment, macrophages in tumor site were repolarized from pro-tumoral M2 to anti-tumoral M1 phenotype, accompanied by the upregulation of IL-12 and the downregulation of IL-10. Moreover, the ratio of CD4^+^ and CD8^+^ T cells was elevated by further combining with αPD-L1, exhibiting strong immune responses (Zeng et al., 2022b). Except for αPD-L1, the synergistic effect is also achieved by combining nanozymes with CTLA-4 blockade. CaO_2_/DOX@SiO_2_/DOX-MnO_2_ (CDSDM) nanozyme reactor was reported with self-oxygenation and alleviation of hypoxia-adenosinergic signaling. Through the positive immune modulation of CDSDM NRs, the infiltration of cytotoxic T cells was facilitated and the population of immunosuppressive regulatory T cells was decreased. On the other hand, the DOX in CDSDM NRs promoted the maturation of antigen-presenting dendritic cells. Thus, the reversion of the immunosuppressive TME is expected to favor CTLA-4-mediated immunotherapy (Wang et al., 2019b).

HA@Cu_2_−xS were synthesized by hyaluronic acid, CuCl_2_ and Na_2_S, which were further modified by PEG to form HA@Cu_2_−xS-PEG nanozymes (PHCNs). The PHCNs nanosystem effectively triggers apoptosis of tumor cells due to its dual photothermal and peroxidase-like catalytic properties. Tumor-specific antigen released from dead tumor cells facilitates the infiltration and activation of chimeric antigen receptor (CAR) T cells, presenting a synergistic effect with CAR T-cell therapy (Zhu et al., 2021).

### Nanozymes in radiotherapy

Radiotherapy occupies an essential position in cancer treatment and has been widely applied for solid tumors in the clinic. However, radioresistance, mainly caused by hypoxia and antioxidant environment around tumor, constrains the efficacy of radiotherapy (Dai et al., 2022). On the one hand, tumor cells overexpress the DNA repair proteins, making them more endurable than normal cells (Roos et al., 2016). On the other hand, the toxicities of the radiation to normal tissues can cause gastrointestinal damage, lung fibrosis, and cognitive impairment in patients, preventing them from being given at high enough doses (Stone et al., 2003). Thus, how to improve the radiation sensitivity of tumors and protect normal tissues from radiation damage has drawn considerable attention (Gong et al., 2022a; Gong et al., 2022b). Therefore, highly selective and multifunctional radiosensitizers are urgently needed for radiotherapy enhancement. To address this issue, nanozymes that can target the tumor and improve hypoxia are promising strategies (Li et al., 2021; Zeng et al., 2021).

The tumor ablation effect of radiotherapy mainly relies on the radiation-induced DNA damage and toxicity of radiation-induced ROS. By coating pyrite (FeS_2_) with cancer cell-derived exosomes, a nanozyme system with homologous targeting abilities, GSH-OXD and POD-like properties was created. The dual enzyme activities of FeS_2_ reduce intracellular GSH levels and generate huge •OH to disrupt redox homeostasis and mitochondria integrity, thus significantly reducing radiotherapy resistance of cancer cells and amplifying the radiotherapeutic efficacy (Huang et al., 2021a).

**FIGURE 5:**
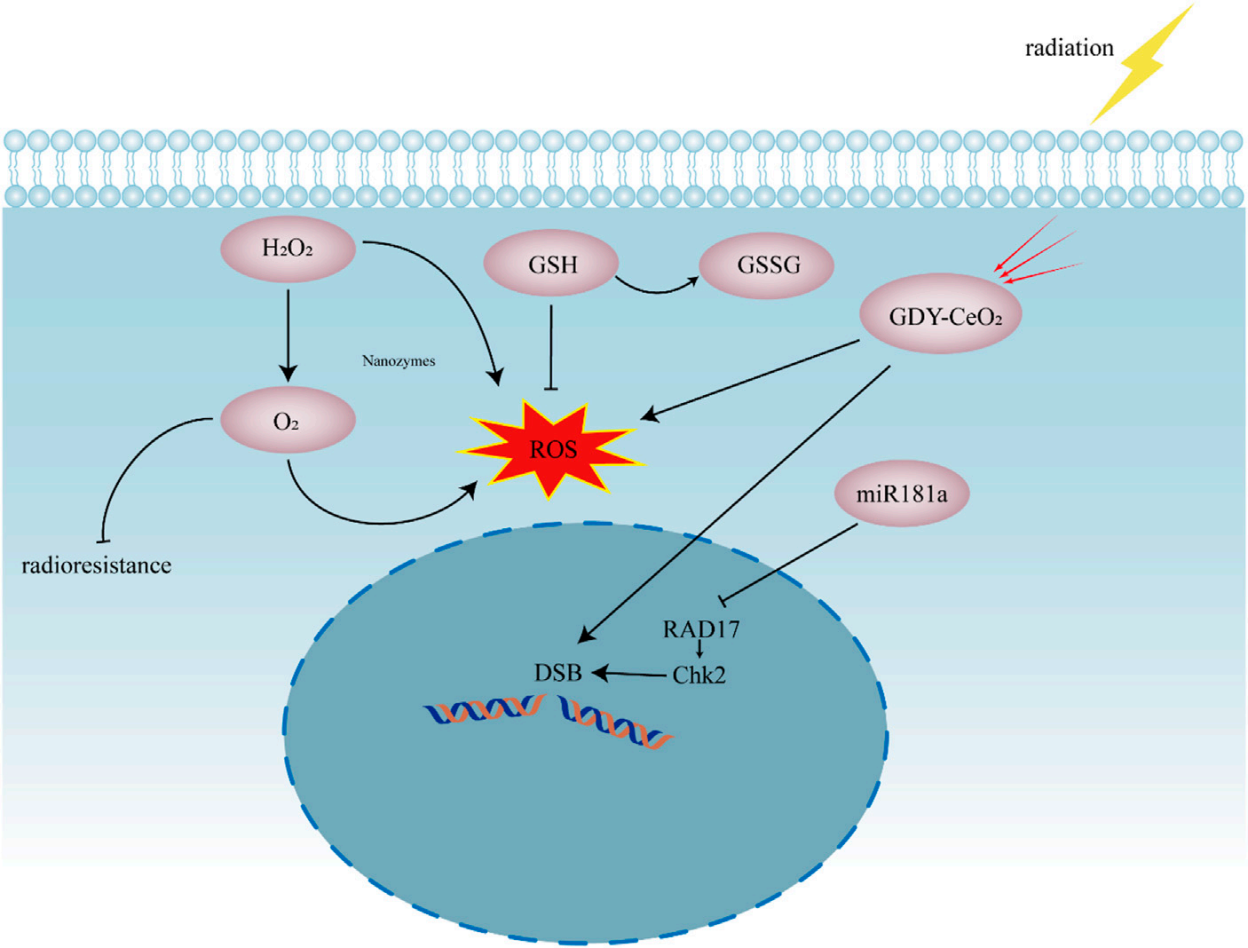
Schematic presentation showing the synergistic effect of nanozymes in radiotherapy. Nanozymes supply O_2_ to reduce radioresistance of tumor cells. The production of ROS enhances the effect of radiotherapy. GDY–CeO_2_ act as radiosensitizer to enhance the intracellular radiation energy deposition. miR181a can regulate RAD17/Chk2 pathway to suppress DNA repair. DSB, DNA double-strand break; GDY, 2D graphdiyne; CeO_2_, Cerium oxide, miR181a, miR181a-2-3p; RAD17, Cell cycle checkpoint protein RAD17; Chk2, Serine/threonine-protein kinase Chk2, ROS, reactive oxygen species; H_2_O_2_, hydrogen peroxide; O_2_, oxygen; GSH, glutathione; GSSG, glutathione disulfide.

GDY–CeO_2_ nanozyme are synthesized by 2D graphdiyne (GDY) anchoring to the Cerium oxide (CeO_2_) nanoparticles. The sustainable catalase activity of GDY–CeO_2_ can decompose H_2_O_2_ and elevate O_2_ concentration in TME, while high-Z element cerium in GDY–CeO_2_ act as a radiosensitizer to enhance the intracellular radiation energy deposition (Figure 5). Moreover, combining miR181a with the GDY–CeO_2_ further regulates the Serine/threonine-protein kinase Chk2 pathway and destroy the DNA repair system in cancer cells, strikingly reinforcing the effect of radiotherapy (Zhou et al., 2021a).

A novel nanoprobes (Mn-doped Ag_2_Se QDs) are engineered by doping Mn (II) ions into Ag_2_Se quantum dot, using Mn (II) ions as CAT mimic to translate H_2_O_2_ into O_2_ and Ag_2_Se QDs as a radiosensitizer and NIR-II fluorescent materials to enhance radiation energy deposition and NIR-II imaging. By conjugating arginine-glycine-aspartate (RGD) tripeptides and polyethylene glycol (PEG) with Mn-doped Ag_2_Se QDs, Mn-doped Ag_2_Se-RGD-PEG nanoprobes were formed showing great biocompatibility and tumor specificity. The specific tumor-targeting and NIR-II-emitting abilities of the nanozymes enable precise NIR-II imaging-guided radiotherapy with negligible damage to normal tissues, which can be applied to boost the efficacy of RT (Wang et al., 2021).

### Nanozymes in chemotherapy

Conventional tumor chemotherapy, which is most commonly applied as first-line therapy for cancer in the clinic, remains a formidable challenge due to the severe side effects and drug resistance (Alkhatib et al., 2022; Zhu et al., 2022a; Wang et al., 2022d; Kang et al., 2022). Through tremendous research by scientists, multifunctional nanozymes are proven to be a promising avenue to achieve higher tumor elimination efficacy in synergistic chemotherapy and catalytic therapy.

On the one hand, nanozymes have the advantage of their enzyme-mimic activities to catalase abundant ROS generation, which cause redox homeostasis disruption and damage to the cell membrane (Yuan et al., 2022). The direct effect of nanozymes can alleviate drug resistance and even kill cancer cells. On the other hand, nanozymes as nanoparticles can encapsulate chemotherapy drugs and release them in the tumor position (Ning et al., 2021). The specific targeting tumor property of nanozymes loaded drug system makes it possible to get better tumor elimination with a lower dose of chemotherapy drugs and less normal tissue injury (Han et al., 2022).

**FIGURE 6:**
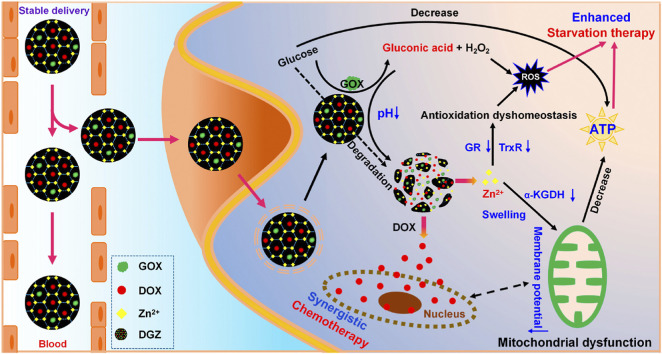
Schematic illustration of the synergistic effect of nanozymes in chemotherapy. Adapted with permission from (Huo et al., 2022). Copyright 2022 American Chemical Society.

Zeolitic imidazolate framework (ZIF)-based nanoparticles co-loaded with glucose oxidase (GOX) and doxorubicin (DOX) were created to synergistically enhance chemotherapy. GOX activity of DOX/GOX-loaded ZIF (DGZ) effectively consumes glucose and generates adequate H_2_O_2_, restraining the mitochondrial energy metabolism and promoting ROS production. As degradation of DGZ, Zn^2+^ was released to break antioxidation homeostasis in cancer cells *via* inhibiting reductase systems such as glutathione reductase and thioredoxin reductase. In case of energy shortage and ROS accumulation, the therapeutic effects of DOX delivered by DGZ get greatly enhanced. More importantly, DGZ releases DOX drug only in the weak acid environment surrounding tumor rather than in normal tissue (Figure 6). This high degree of targeting makes it possible to use higher doses of chemotherapy drugs to get better therapeutic outcomes with fewer side effects (Huo et al., 2022).

The hypoxia-related drug resistance and the lack of potential uptake of drugs also hamper chemotherapy application in tumor treatment. Zheng et al. fabricated Fe_3_O_4_/Pt-FLU nanozymes by loading Pt nanozymes and 5-fluorouracil on the Fe_3_O_4_ nanospheres to achieve improvement in the treatment of breast cancer. The POD-liked and CAT-liked activities of Fe_3_O_4_/Pt-FLU alleviate the hypoxia environment in TME by increasing the level of ROS and O_2_, which leads to the reduction of drug resistance of cancerous tissue. Furthermore, Fe_3_O_4_/Pt-FLU specifically delivers 5-fluorouracil to the tumor site in a pH-sensitive drug release manner. The drug carrier role of Fe_3_O_4_/Pt-FLU significantly increases the stability of 5-fluorouracil and reduces the cytotoxicity in off-target tissues. The catalytic/chemotherapy double-modality therapy shows great therapeutic outcome for tumor elimination (Nie et al., 2022).

### Nanozymes in sonodynamic therapy

Ultrasound (US)—triggered sonodynamic therapy (SDT) is mainly based on low-intensity US and sonosensitizers, which can induce the generation of cytotoxic ROS for tumor elimination (Chen et al., 2021a; Huang et al., 2021b). Compared with traditional therapy, SDT has the advantage of noninvasiveness, high tissue-penetrating capability, and safety, showing promising applications in new generation antitumor treatment (Pan et al., 2018; Son et al., 2020; Sun et al., 2021a). However, the ROS yield of sonosensitizers requires adequate O_2_ assistance which is strongly hampered by hypoxia of TME. Thus, synergistic therapy with nanozyme-based O_2_ supply and SDT may be a practical strategy (Gong and Dai, 2021).

AIMP, a multifunctional nanozyme, is formed by linking Angiopep-2 (Ang) with PLGA, in which IR780 and MnO_2_ are encapsulated. Through the function of Ang and IR780, AIMP can target cancer cells and remain inside the mitochondria. Upon low-intensity US irradiation, IR780 behave as sonosensitizer to produce ROS and cause damage to cancer cells. On the other hand, enzyme-like activity of MnO_2_ is responsible for O_2_ release and GSH depletion, thereby boosting the production of ROS and enhancing SDT effect (Liu et al., 2021b).

**FIGURE 7:**
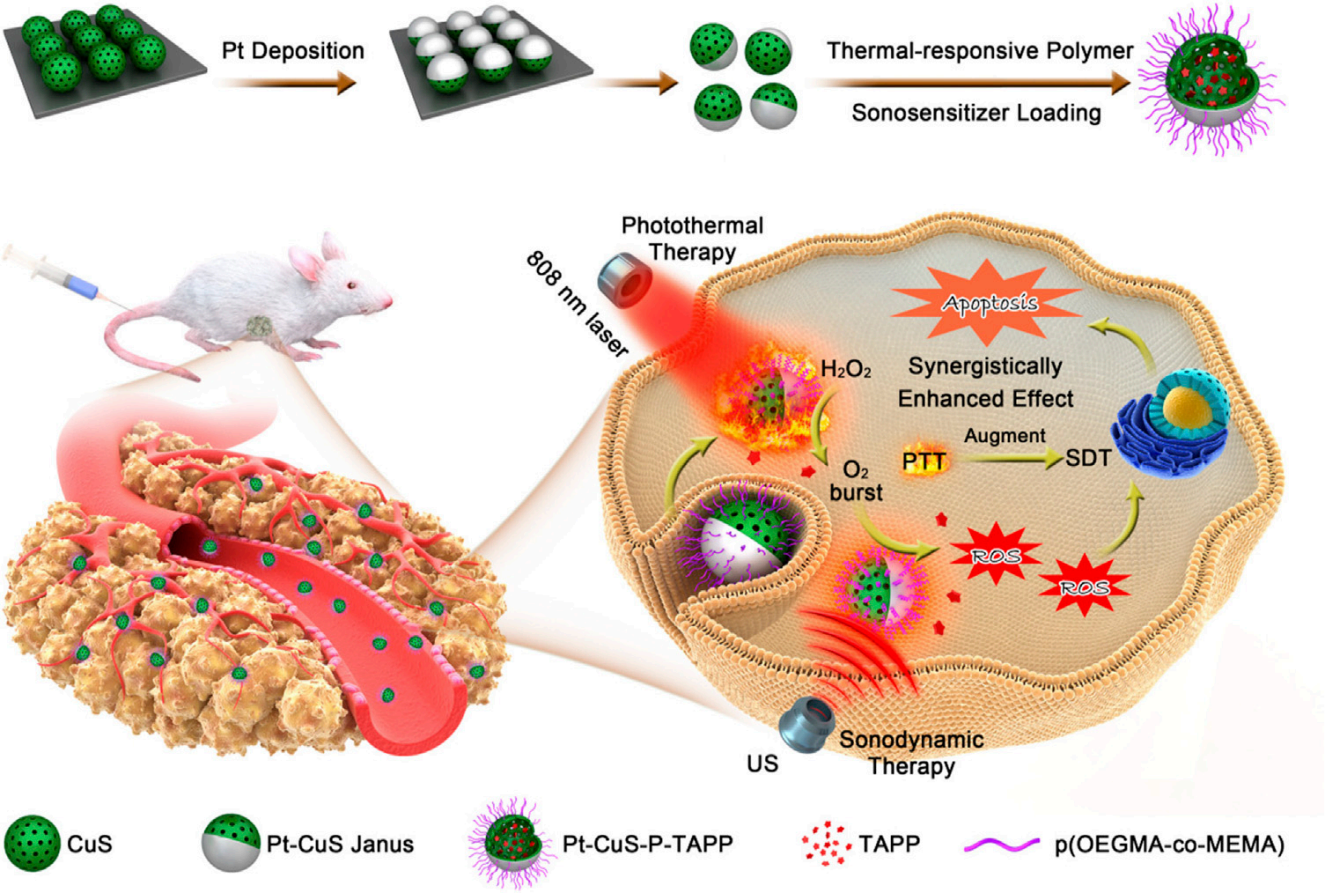
Schematic illustration of the synergistic effect of nanozymes in sonodynamic therapy. Adapted with permission from (Liang et al., 2019). Copyright 2019 American Chemical Society.

The Pt-CuS Janus loaded with tetra-(4-aminophenyl) porphyrin (TAPP) as a sonosensitizer is designed to facilitate SDT-based synergistic therapeutic modality. Upon exposure to US, TAPP are released from the hollow Pt-CuS Janus and interact with O_2_ to produce ^1^O_2_. Meanwhile, Pt possesses CAT-like activity for the degradation of H_2_O_2_ and production of O_2_, which provides an O_2_ source for SDT-induced ROS production (Figure 7). Importantly, the photothermal effect of Pt-CuS elevates the Pt catalytic activity for O_2_ generation and facilitates SDT efficacy (Liang et al., 2019).

### Nanozymes in chemodynamic therapy

Chemodynamic therapy (CDT) is an emerging therapeutic strategy that can destroy cancer cells through catalyzing the endogenous H_2_O_2_ into lethal hydroxyl radical by Fenton reaction or Fenton-like reaction (Zhou et al., 2021b; Jia et al., 2022). CDT takes advantage of high selectivity, low side effects and no requirement for exogenous stimulus. However, the limited concentration of endogenous H_2_O_2_ and excessive antioxidant GSH hinder the application of CDT. Supplying sufficient intratumoral H_2_O_2_ and depleting endogenous GSH are necessary to improve the efficiency of CDT. Recently nanozyme cascade platforms have been extensively studied and wildly applied to enhance the antitumor effect of chemodynamic therapy (Zhang et al., 2021; Zhang et al., 2022e).

**FIGURE 8:**
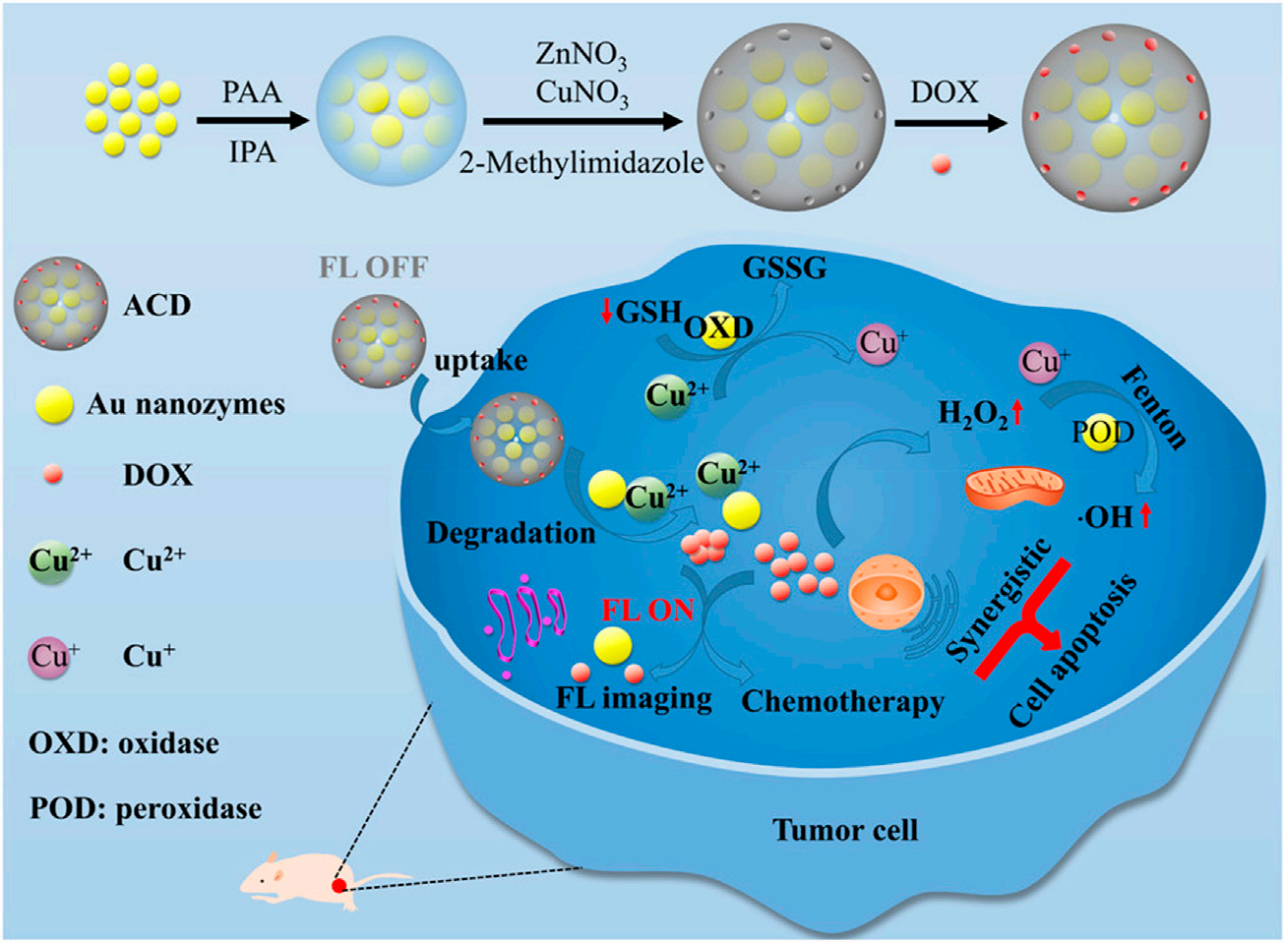
Schematic illustration of the synergistic effect of nanozymes in chemodynamic therapy. Adapted with permission from (Zhao et al., 2021a). Copyright 2021 American Chemical Society.

The cascade reaction systems ACD is fabricated by loading copper ion-doped ZIF-8 with ZIF-8 doped with Au nanozymes and doxorubicin hydrochloride (Zhao et al., 2021a). The POD-like activity of Au nanozymes can catalyze H_2_O_2_ to ·OH for CDT. While Au can also serve as an oxidase to deplete the GSH. Moreover, the Cu^2+^ ions released from ACD could consume the GSH *via* redox reactions and the generated Cu^+^ can produce ·OH by Fenton-like reaction. Thus, the increased ROS and exhaustion of GSH can effectively amplify the antitumor effect of CDT. On the other hand, doxorubicin hydrochloride not only act as chemotherapeutic drug to kill cancer cells but also supply sufficient H_2_O_2_ to boost the effect of CDT (Figure 8). Herein, the application of nanozymes in CDT can achieve better treatment effects and are promising for tumor elimination.

Apart from increasing ROS production and promoting GSH depletion, strengthening the tumor targeting ability of nanodrug is another way to augment the effect of CDT. Au-DNA-Fe, a microRNA-triggered nanozyme cascade platform is fabricated for enhanced tumor-specific chemodynamic therapy. Au nanozyme as glucose oxidase-like nanozyme catalyze Glucose into gluconic acid and H_2_O_2_, while the produced H_2_O_2_ is subsequently catalyzed by Fe_3_O_4_ nanoparticles to generate hydroxyl radicals for CDT. Interestingly, Au and Fe_3_O_4_ nanoparticles are modified by two different single-stranded DNA which are complementary to each part of miRNA-21. Due to the overexpression of miRNA-21 in cancer cells, the nanozyme Au-DNA-Fe can trigger the cascade reaction specifically in cancer cells with negligible side effects on normal tissues (Liu et al., 2021a).

## Conclusion and perspectives

Nanozyme is a breakthrough in the treatment of cancer, and their extremely small size, good modifiability and applicability provide endless opportunities to design multi-program cancer therapy strategies. A lot of research has generated a large number of feasible solutions for nanozymes involved in cancer treatment, mainly *via* inducing oxidative stress by catalyzing ROS production and disrupting the function of tumor cells by altering the metabolism. The imbalance in redox homeostasis caused by excessive ROS induces apoptosis or ferroptosis, which both result in tumor death. In addition to the direct killing of tumor cells, modification of the tumor microenvironment by nanozymes effectively reduces the resistance of tumor cells to therapy, including drug resistance, radiation desensitization, or immune evasion. This creates an amazing synergy when used in conjunction with other classical therapies such as phototherapy, immunotherapy, radiotherapy, and chemotherapy.

Despite the promising future, many hurdles remain to be overcome in the application of nanozymes in cancer therapy. Oxidases, catalase and hydrolases already exist in numerous applications in the treatment of cancer, but the study of other enzymes such as transferases and lysates are new direction and relatively empty areas of research. Exploration of novel enzymatic nanoparticles can help us establish a multi-enzyme nanoparticle therapeutic system. Due to their diverse catalytic activities, different enzyme types can synergistically address the difficulties that exist in aberrant tumor environments and play a more effective role in tumors.

On the other hand, the catalytic activity of nanozymes needs to be improved. Although there is a small subset of nanozymes with greater catalytic activity than natural enzymes. However, achieving optimal catalytic efficiency in a complex tumor microenvironment remains a challenge. Most importantly, good biocompatibility is a pre-requisite for nanozymes to be used in the clinic. There is an urgent demand to reduce nanozymes inherent toxicity or design nontoxic nanozymes, for which ensuring that nanozymes remain effective at low doses is a viable method. Thus, it is the key to improving the targeting ability of nanozymes, as well as the ability to deliver them into the bloodstream in a sustained manner. The construction of a nanozymes-system with good biocompatibility and high target efficiency will be future development trend in new generation cancer therapy.

As nanotechnology continues to develop and cancer treatment improves, nanozymes with high catalytic efficiencies, excellent biocompatibility, specificity, and multiple functionalities in combination with traditional anticancer therapy are expected to emerge in the future.
